# Gender differences in professional drivers’ fatigue level measured with BAlert mobile app: A psychophysiological, time efficient, accessible, and innovative approach to fatigue management

**DOI:** 10.3389/fpsyg.2022.953959

**Published:** 2022-08-01

**Authors:** Ricardo De La Vega, Hector Anabalon, Kyran Tannion, Helena Purto, Cristian Jara D

**Affiliations:** ^1^Physical Education, Sport and Human Movement, Autonomous University of Madrid, Madrid, Spain; ^2^AlertPlus, Santiago de Chile, Chile; ^3^Department of Neurology, Pontificia Universidad Católica de Chile, Santiago, Chile

**Keywords:** app, heart rate variability, mental fatigue, Stroop effect, gender

## Abstract

Addressing fatigue is useful in a variety of scenarios and activities. Fatigue has recently been studied from a psychophysiological standpoint. As a result, the expression and impact of peripheral and central fatigue has been evaluated. Driving is one occupation where tiredness has disastrous consequences. BAlert is a smartphone app that approaches exhaustion with psychophysiological measures. More specifically, it evaluates the level of fatigue *via* heart rate variability (HRV) data and the cognitive compromise *via* Stroop effect. The goal of this study is to determine if there are gender differences in fatigue levels among professional drivers using the BAlert app. Statistically significant differences were found in the number of hours awake, in different parameters of HRV (AVNN, PNN50, RMSSD, and SDNN), in the level of stress, as well as in the cognitive response evaluated through the app. The results are discussed and their implications for the management of work fatigue are presented.

## Introduction

Understanding the psychophysiological effects of internal and external demands on the development of fatigue is essential (Holm et al., 2009; Du et al., 2020; De La Vega et al., 2021a). As fatigue has become increasingly relevant in a variety of contexts, it has gained attention in recent years (Brown, 1994; Enoka and Duchateau, 2016). Fatigue as an outcome depends on an individual’s tolerance level and ability to adapt. In other words, it is a result of a load that surpasses the skills and coping strategies of the person facing that demand (DeLongis et al., 1988; Folkman and Lazarus, 1988). Explanation of fatigue due to external loads requires both central and peripheral factors to be considered (Davis, 1999; Meeusen et al., 2006; Amann, 2011; Zaja̧c et al., 2015). Central and peripheral factors are task-dependent. Fatigue in the central nervous system may be linked to task failures, while peripheral fatigue may appear early when external load demands are high (Decorte et al., 2012; Thomas et al., 2015). It is noteworthy that external demands that are of significant intensity can lead to a high level of respiratory muscle work, which in turn causes peripheral fatigue of locomotor muscles (Romer et al., 2006). Furthermore, internal demands may also lead to significant changes in respiratory function (Grassmann et al., 2016). Even more importantly, psychophysiological responses that follow an external demand can be obtained at a similar level when an individual is subjected to an internal demand (i.e., imagery) of equivalent intensity (Wang and Morgan, 1992). Due to the lack of economic, attainable, effective and direct measures, the central fatigue assessment has been overlooked when it comes to the mental component. On the other hand, physical fatigue assessment procedures have been developed successfully (Mehta and Parasuraman, 2014; Monteiro et al., 2019; Dotan et al., 2021; Díaz-García et al., 2021).

Demanding tasks, whether physical or cognitive, can lead to homeostatic imbalance and poor performance in a variety of situations (Barker and Nussbaum, 2011; Smith et al., 2016; Van Cutsem et al., 2017; Pageaux and Lepers, 2018). Moreover, when the expected reward value is outweighed by the perceived homeostatic imbalance (i.e., energy cost or tiredness), an individual is no longer encouraged to engage in task performance (Boksem and Tops, 2008). In a dynamic environment making decisions correctly relies on sustained attention to process information (Walsh, 2014), and mental fatigue may appear when engaging in enduring cognitive efforts (Marcora et al., 2009). In light of the foregoing, it is clear that accurate and precise assessment of central fatigue on the mental dimension, not just peripheral, is critical. The Stroop effect could be beneficial in delivering an objective and reliable measure of mental exhaustion. When two stimuli of different dimensions are presented simultaneously, one of which is required to accomplish the task correctly (vs. the other which is irrelevant to the task), an interference effect occurs (i.e., Stroop effect, Banich, 2019). It is important to note that the Stroop task has traditionally been employed to create mental tiredness (e.g., Smith et al., 2016; Veness et al., 2017; Penna et al., 2018; Gantois et al., 2020) rather than assess it (e.g., Verschueren et al., 2020).

In the context of physical activity and sports, a variety of heart rate variability (HRV) measures have been widely and efficiently utilized to evaluate fitness and fatigue responses to a number of training loads (Buchheit et al., 2004; Kaikkonen et al., 2012; Wallace et al., 2014). Not just throughout physical activity, but also during rest and recovery, such as days off from the training plan. In that scenario, resting Ln rMSSD is a strong HRV fatigue sign (Djaoui et al., 2017). The focus of research has shifted away from first-generation questions (e.g., “Is HRV capable of monitoring fatigue?”) and toward second-generation questions (such as “What HRV parameters have more potential to evaluate fatigue?”). All in all, the Stroop effect isn’t the only objective measure of exhaustion (i.e., central and peripheral) that holds promise. Furthermore, not only has HRV proven to be a valid and accurate metric for assessing physical fatigue, but it has also shown reliability and validity as a metric for assessing mental exhaustion (e.g., Egelund, 1982; Laborde et al., 2011; Melo et al., 2017; Anwer et al., 2021; Qin et al., 2021). According to Huang et al. (2018), the following HRV parameters are the most significant markers (i.e., sensitive): NN.mean (mean of normal to normal interval), PNN50 (percentage of NN50 divided by total number of NNs), TP (total spectral power), and LF (low frequency from 0.04 to 0.15 Hz). Additionally, high-frequency HRV appears to be sensitive to emotional regulation and trait and state coping-related variables (Laborde et al., 2015). Overall, the Stroop effect and HRV parameters appear to be appropriate, reliable, and effective tools for tiredness and stress monitoring (De La Vega et al., 2021b).

There are sex-specific differences in cardiovascular autonomic regulation (Taylor et al., 2020). Furthermore, biological sex plays a key role in the neuroendocrine modulation of the cardiovascular system. This could be given by both anatomical and physiological differences between males and females, and most probably the cardioprotective effects of estrogen (Prabhavathi et al., 2014). Since HRV is a non-invasive tool, it is a useful way of measuring autonomic function, but at the same time it is also subject to the factors that influence autonomic control. Gender differences in HRV have been previously reported (Bonnemeier et al., 2003), therefore it is essential that we deepen our understanding of them. This way, we can further improve the sensitivity of fatigue detection through cardiovascular parameters together with other variables.

As previously discussed, fatigue knows no limits when it comes to human activities (Enoka and Duchateau, 2016). Driver drowsiness warrants special attention because it plays a significant role in traffic accidents and safety (Brown, 1994; Lal and Craig, 2001; Zhang et al., 2016). Many metrics have demonstrated to be valid in assessing drivers’ cognitive states. Electroencephalography (EEG) and event-related potentials (ERP), optical imaging for cerebral blood flow, blood pressure (BP), electromyography (EMG), thermal imaging, HRV, and pupillometry are only a few examples (Lohani et al., 2019). HRV monitoring technology is critical in the early diagnosis of alertness and weariness in drivers. Their evaluation and quantification are required to reduce the dangers connected with a weary operator’s irregular performance (Egelund, 1982; Patel et al., 2011; Lin et al., 2018). Future research and practical technologies implementation faces the difficulty to suggest a fatigue management strategy that is time efficient, affordable, and objective. Smartphones and app development appear to have a lot of potential to withstand such a challenge. Mobile Health (mhealth) is a reality with a bright future in the treatment of pain, psychological distress, weariness, and sleep (Hernandez Silva et al., 2019). There are currently apps that use self-report and test measurements to detect fatigue in clinical populations (Mäcken et al., 2021). In addition, real-time facial and eye tracking is being used in drivers (Abulkhair et al., 2015). BAlert is a pioneering tool in terms of implementing objective psychophysiological fatigue metrics through a smartphone app (De La Vega et al., 2021b).

Despite the great value of these apps, more research is needed to ensure that they continue to provide real benefits to their users. As a result, the primary goal of this study was to determine if there are gender disparities in fatigue parameters among professional drivers using the BAlert app.

## Materials and methods

### Experimental design and participants

This study followed a quasi-experimental design (Montero and León, 2007). The tasks were fulfilled by a total of 2,331 professional drivers (i.e., BAlert). It took 2 min to accomplish both tasks. The sample was of 2,252 men (96.6%) aged 44.26 ± 10.997 and 79 women (3.6%) aged 36.11 ± 8.5. It’s important to consider that in this labor sector the participation of women is significantly lower than that of men. This justifies the percentage difference between them, an effect offset by a large sample number. For the collection of the sample, the voluntary help of the workers of the main mining and transport companies in Chile was requested, ensuring the confidentiality of the data and agreeing to collaborate jointly with the human resources departments of each of the companies.

### Instrumentation and study variables

The BAlert app, which can be downloaded for Android and iOS, was used to measure all of the variables in the study. The engineering team made upgrades to the earlier version (i.e., De La Vega et al., 2021b) taking into account the suggestions made by the investigators. Attempts were conducted on beta releases of the software to ensure that the updated features worked as intended. The app provides participants reports (i.e., output) on their level of fatigue based on the responses or results of each task (i.e., input).

1. *Hours awake.* Self-reported.
2. *Stroop Color-Word test.* The number of correct responses in a predetermined period of time is recorded. The test’s scoring was based on a proposal by Scarpina and Tagini (2017).
3. *Temporal parameters of the Heart Rate Variability.* Especifically, AVNN, RMSSD, SDNN, and PNN50.
4. *Samn-Perelli scale and Epworth questionnaire*. According to Gawron’s (2016) overview, they have proven to be appropriate and adequate for the scenario (2016). It is worthy to mention that the Samn-Perelli scale is modified and goes from 1 to 10.
5. *Stress*. Based on the inputs it is reported by the apps software as a continuous variable.
6. Stroop output, PNN50 output and *overall fatigue level.* Based on the inputs, software reported as categorical variables (i.e., severe, normal, moderate).

Due to company confidentiality, the procedure for obtaining software reported values can’t be revealed. BAlert is indeed a work-in-progress app. As a result, the company retains its rights.

### Procedure

The research’s goal and procedure were explained to each participant. The participants downloaded the software onto their personal smartphones. Participants supplied their informed consent and completed the proposed tasks once.

### Statistical analysis

The Kolmogorov-Smirnov test was used to determine whether the variables were normal. None of the variables had a normal distribution (*p* < 0.05). The Chi-squared test was performed to determine whether categorical variables were independent. To identify significant differences between groups, the Mann-Whitney *U*-test was employed. The effect size was determined using the Fritz et al. (2012) guidelines.

## Results

Chi-square test proved there was dependence (*p* < 0.05) between overall fatigue level and gender (i.e., male vs. female), PNN50 output (i.e., severe, normal, moderate) and Stroop output (i.e., severe, normal, moderate), respectively. Also between gender and PNN50 output and Stroop Color-Word test output. Also between Stroop Color-Word test output and PNN50 output. Table 1 shows gender differences in all the fatigue parameters we evaluated (i.e., Hours awake, HR, HRV, Stroop Color-Word test, Samn-Perelli Scale, Epworth questionnaire and Stress). HRV values (AVNN, PNN50, RMSSD and SDNN) were higher in females than males in this study, as seen in Table 1.

**TABLE 1:** Gender differences in fatigue parameters.

Study variable	Men (mean ± SD)	Women (mean ± SD)	*p*	Effect size
Hours awake	2.76 ± 1.73	**4.06 ± 2.08**	0.000*	0.015
HR (bpm)	**78.23 ± 12.86**	74.43 ± 9.481	0.005*	0.003
HR (%)	77.33 ± 19.65	77.43 ± 19.19	0.915	0.000
AVNN	789.46 ± 133.35	**819.28 ± 101.55**	0.006*	0.003
PNN50	15.34 ± 17.73	**22.76 ± 20.98**	0.001*	0.005
RMSSD	35.42 ± 21.02	**43.39 ± 23.41**	0.000*	0.005
SDNN	39.01 ± 18.32	**43.73 ± 17.77**	0.009*	0.003
Stroop color-word test	**27.98 ± 5.08**	23.89 ± 8.23	0.000*	0.009
Samn-Perelli scale	**8.86 ± 1.53**	7.05 ± 3.15	0.000*	0.013
Epworth questionnaire	0.64 ± 1.69	**2.25 ± 3.91**	0.000*	0.006
Stress	403.93 ± 284.007	314.80 ± 194.510	0.009*	0.003

Bold values indicate statistical significance at the p < 0.05 level. Values are expressed as means ± standard deviation. *p < 0.05.

## Discussion

The results found regarding the differences in the parameters of HRV and HR indicate that females have a greater resting heart rate than men. Several studies report that women have a greater parasympathetic activity on the heart, while men have a sympathetic dominance (Koenig and Thayer, 2016). We found gender-specific differences in HRV parameters and HR, but these differences cannot be solely attributed to gender, since there wasn’t standardization of other variables like age (Umetani et al., 1998) or physical activity, two of the many factors that impact HRV and HR.

Heart rate variability is considered a visceral signature of both body state and mental activity (Hansen et al., 2003). This can be understood by looking at bidirectional (top-down and bottom-up) connectivity between cortical areas such as the prefrontal cortex and more visceral regions in charge of emotional and autonomic responses. This neurovisceral integration (Thayer and Lane, 2009) allows us to perceive and respond appropriately to external and internal signals (Critchley et al., 2013). From a psychophysiological perspective, it is certainly possible and expected that a Stroop task would show dependence on a HRV parameter such as PNN50. These findings add to the previous body of evidence associating HRV and cognitive performance (Hansen et al., 2003).

Loss of inhibitory control over stress-responsive neural structures is shown to result in a perturbation of autonomic outflow characterized by reduced vagal tone (reflected in low HRV) and heightened stress reactivity (Beaumont et al., 2012). Fatigue-inducing lifestyle habits and psychosocial background and environment contribute to the emergence of fatigue in the individual. This eventually weakens the ability to tolerate stress, hardships and unpredictable life experiences, which furthermore contributes to the experienced fatigue. This downward spiral occurs in part because during fatigue, and especially stress-induced fatigue, there aren’t enough cognitive resources available for the top-down regulation from the prefrontal cortex that guides our behavior, emotional regulation, guidance of thought, and attention (Arnsten, 2009).

It is noteworthy that men and women engage different neural circuitry patterns in response to stress. The ventromedial prefrontal cortex has inhibitory projections toward the amygdala, which may be in part responsible for the ability to cope with stressors (Goldfarb et al., 2019). On the other hand, loss of the inhibitory control over these structures involved in the stress response has shown to result in a reduced vagal tone and therefore lower HRV (Beaumont et al., 2012). Ultimately this explains how HRV can be used to assess stress levels and ability to cope.

There are gender specific differences in the stress response, specifically in certain arousal systems; for example, the female Locus Coeruleus—Norepinephrine arousal system has a stronger and persistent stress response than the male arousal system (Bangasser et al., 2019). There are gender differences both in the response and in the regulation of stress (i.e., resilience). Therefore, the differences we see in the Stress Index (Baevsky and Chernikova, 2017) between males and females are to be expected. Nevertheless, these differences may be explained not only by gender, but also activity levels (which impact resting heart rate upon which the Stress Index is calculated), social background, education, and other factors that influence the human stress response. In the future we hope to assess these differences with a more balanced sample size of women, to further investigate and elucidate these findings. We expect the neurophysiological gender-specific differences in stress response will be further studied, to better estimate stress parameters and improve stress management. Gender-differences in stress response have implications not only in fatigue management but also in mental health, which ultimately also impacts fatigue levels.

Sleep is a central element in the development of fatigue. It is well known that on average women require more hours of sleep than men (Burgard and Ailshire, 2013). Loss of sleep or sleep fragmentation has a negative impact on overall cardiovascular health. A single night of poor quality sleep can be quantified through changes in HRV values.

In this manner, measuring cognitive ability through the Stroop test together with HRV has proven to be an effective method of fatigue detection and management (De La Vega et al., 2021a). This novel method integrates different domains through which fatigue manifests itself: firstly, the cognitive impairments that occur when fatigue arises can be detected through the Stroop test; and secondly, the physiological manifestation of the autonomic balance of the individual can be measured through HRV which is in an of itself an indicator of wellbeing and homeostatic balance. Furthermore, the BAlert tool bypasses the need for subjective fatigue measurement (i.e., self-report) and therefore isn’t tied to the ability of the individual to self-monitor their fatigue levels (i.e., interoception). Instead, BAlert implements objective and quantifiable parameters that are connected to and arise from neurophysiological interoceptive processes (i.e., Neurovisceral Integration Model).

We have not only seen that fatigue manifests itself in a different manner between males and females, but also that environmental fatigue-inducers could affect men and women in different ways.

## Data availability statement

The database belongs to the company AlertPlus S.A. This company undertakes to attest to the veracity of the data presented. Anyone who wishes to access the data must make a formal request through the web: https://www.alertplus.net/, including the reasons for the request. AlertPlus S.A. reserves the right to transfer the data.

## Ethics statement

This study was developed according to the Standards for Ethics in Sport and Exercise Science Research (Harriss et al., 2019). It was approved by the University Ethical Commission before being carried out, in accordance with the Helsinki Declaration (CEI-106-2060). The goal, approach, and process were all explained to all of the participants. Finally, they signed a consent form indicating their willingness to participate.

## Author contributions

RD designed and coordinated the research and collected the data. HA, HP, and KT helped in the design, data collection, data-recording, and data-verification. CJ and KT participated in writing, literature search, and formatted the manuscript for publication. RD, KT and HP performed the statistical analyses and wrote the “Materials and methods,” “Discussion,” and “Conclusion” sections of the report. All authors contributed to the article and approved the submitted version.
